# Automatic Classification of Thermal Phases During Steel Oxidation via PCA and Infrared Thermography Time Series

**DOI:** 10.3390/ma19173699

**Published:** 2026-08-31

**Authors:** Antony Morales-Cervantes, Gerardo Marx Chávez-Campos, Héctor Javier Vergara-Hernández, Jorge Sergio Téllez-Martínez, Luis Ulises Chávez-Campos, Mayra Yunuen Rincón-Pineda, Edgar Guevara

**Affiliations:** 1 TecNM-Instituto Tecnológico de Morelia, Av. Tecnológico 1500, Morelia 58120, Mexico; antony.mc@morelia.tecnm.mx (A.M.-C.); gmarx_cc@itmorelia.edu.mx (G.M.C.-C.); hector.vh@morelia.tecnm.mx (H.J.V.-H.); jorge.tm@morelia.tecnm.mx (J.S.T.-M.); luis.ulises.cc@morelia.tecnm.mx (L.U.C.-C.); mayra.rp@morelia.tecnm.mx (M.Y.R.-P.); 2 Faculty of Science, Universidad Autónoma de San Luis Potosí, Sierra Leona 550, San Luis Potosí 78120, Mexico; 3 Coordinación para la Innovación y Aplicación de la Ciencia y la Tecnología-CIACYT, Universidad Autónoma de San Luis Potosí, Sierra Leona 550, San Luis Potosí 78120, Mexico

**Keywords:** high-temperature oxidation, AISI 1045 steel, infrared thermography, image processing algorithms, thermal phase classification, steel manufacturing

## Abstract

High-temperature oxidation affects the surface condition and quality of steel products, yet automated identification of the thermal stages governing oxide-scale formation remains limited. This study evaluated whether infrared thermography time series combined with dimensionality reduction and supervised learning could classify the heating, isothermal, and cooling phases of AISI 1045 steel oxidation. Five controlled Joule-heating experiments generated 3356 thermographic records. Each thermogram was flattened, and principal component analysis was applied independently to each experiment; the first two principal components were then used to train Random Forest, Multi-Layer Perceptron, Support Vector Machine, k-Nearest Neighbors, and XGBoost classifiers. Performance was assessed using leave-one-group-out cross-validation to preserve specimen-level independence. The Multi-Layer Perceptron achieved the best performance, with an accuracy of 0.8934, Macro-precision of 0.8740, Macro-recall of 0.8938, and Macro-F1-score of 0.8562. The highest class-specific area under the receiver operating characteristic curve was 0.95 for cooling. Most errors occurred between temporally adjacent phases, whereas confusion between heating and cooling was negligible, indicating physically consistent classification. These findings establish a proof of concept for automated thermal-phase recognition from raw thermographic sequences and support further validation under broader materials, processing conditions, and industrial environments.

## 1. Introduction

High-temperature oxidation is intrinsic to several steel production and thermal-processing routes, including reheating, hot rolling, and heat treatment, where the surface is exposed to aggressive atmospheres at elevated temperatures. The resulting oxide scales influence scale-growth kinetics, surface condition, and downstream product quality, while their development depends strongly on temperature, gas composition, and flow conditions [1]. Oxide-layer growth typically follows mixed linear and parabolic kinetics governed by transport phenomena and interfacial reactions, which are highly sensitive to processing conditions.

Infrared thermography (IRT) has emerged as an powerful tool for monitoring such processes due to its non-contact, spatially resolved, and real-time measurement capabilities, particularly in environments where conventional sensors are impractical or intrusive [2,3]. In metallurgical applications, IRT has demonstrated the ability to characterize high-temperature phenomena in situ. For example, it has been successfully employed to analyze solidification behavior and spatial thermal evolution in steels, revealing distinct thermal signatures associated with phase transformations and material heterogeneity [4].

Despite these advantages, the interpretation of thermograms during oxidation remains challenging. The recorded radiative signal not only depends on temperature, but also on emissivity, which evolves dynamically with oxidation time, oxide-layer thickness, and temperature. In carbon steels, oxidation induces significant variations and even oscillatory behavior in spectral emissivity due to thin-film interference effects [5]. These emissivity fluctuations can introduce substantial errors in temperature estimation if not properly accounted for, but they also encode valuable information about surface transformations. Recent studies have leveraged this sensitivity in AISI 1045 steel, enabling real-time detection of oxide-layer formation and quantification of spatiotemporal thermal heterogeneity through advanced image-processing techniques [6,7]. These findings demonstrate that thermographic time series contain rich descriptors of oxidation dynamics beyond conventional temperature tracking.

The next methodological step is to transform these dense radiometric sequences into automated and reproducible decision workflows. Machine learning approaches are increasingly being integrated into thermographic and image-based materials inspection to accelerate interpretation and enable data-driven monitoring [8,9,10]. Recent studies in electron/laser beam processing and silicon carbide wafer inspection have shown that deep-learning models can improve the recognition of weak-edge features, micro-defects, and complex surface patterns in manufacturing environments [9,10]. However, the high dimensionality of thermal image time series necessitates efficient feature extraction strategies that preserve the dominant thermal structures. Principal component analysis (PCA) provides a well-established method for dimensionality reduction by projecting data onto orthogonal components that capture the largest variance [11]. In thermographic applications, PCA has proven effective in extracting representative features from infrared image sequences, enhancing relevant thermal patterns while reducing redundancy and noise [12].

Within this context, the automatic discrimination of the global thermal phases that define a high-temperature oxidation cycle (namely heating, isothermal exposure, and cooling) remains comparatively underexplored. Existing studies have primarily focused on oxide detection or qualitative thermal analysis rather than on the segmentation of the process into physically meaningful states. A robust phase classification workflow would convert continuous thermographic acquisition into discrete and actionable process states, enabling objective tracking of thermal history and establishing a basis for anomaly detection and adaptive control strategies.

Compared with previous infrared-thermography studies on AISI 1045 oxidation, which focused mainly on oxide-layer detection and monitoring [6], spatiotemporal thermal heterogeneity [7], or apparent oxidation-kinetics descriptors [13], the novelty of the present work lies in the automatic classification of the global thermal stages of the oxidation cycle. Specifically, this study transforms raw thermographic time series into experiment-wise PCA trajectories and evaluates supervised classifiers under specimen-aware validation to distinguish heating, isothermal exposure, and cooling as physically meaningful process states. To address this gap, this work proposes a PCA-based workflow for the automatic classification of thermal phases during high-temperature oxidation of AISI 1045 steel using raw infrared thermography time series. By combining dimensionality reduction with supervised learning under specimen-aware validation, the proposed approach aims to provide a reproducible and physically interpretable methodology for in situ process monitoring.

## 2. Materials and Methods

### 2.1. Experimental Setup

**Figure 1:**
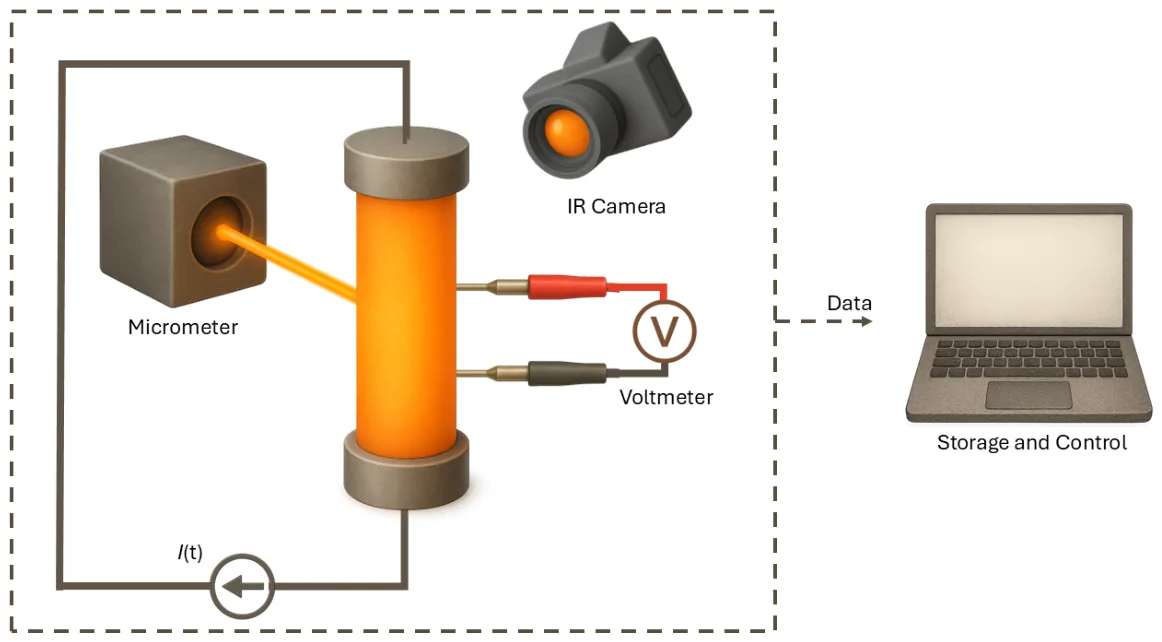
Configuration of the experiment for the acquisition of images by infrared thermography.

Five high-temperature oxidation experiments were conducted on cylindrical 1045 steel specimens with a diameter of 3.8 mm and a length of 26 mm. AISI 1045 is a medium-carbon steel with a typical nominal composition of approximately 0.42–0.50 wt.% C, 0.60–0.90 wt.% Mn, ≤0.040 wt.% P, ≤0.050 wt.% S, and Fe as the balance [14]. The oxidation process was carried out in a controlled Joule heating system. The experiment lasted a total of 50 min, with temperatures exceeding 700 °C. The heating of the specimen was performed incrementally, with the current increased by 16 amperes every minute until reaching 160 amperes. This current was maintained for 30 min to analyze the surface changes induced by the oxide layer formation. Finally, the specimen was cooled down gradually, decreasing the current by 16 amperes every minute until reaching 0 amperes. The setup for infrared image acquisition during the heating of 1045 steel is depicted in Figure 1.

**Figure 2:**
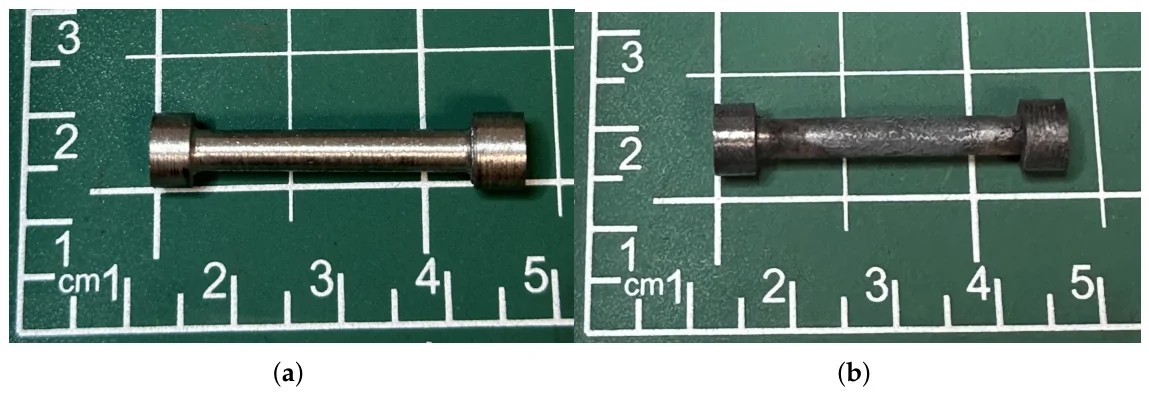
Representative optical images of AISI 1045 steel specimens used in the Joule-heating experiments: (**a**) initial machined specimen before oxidation and (**b**) oxidized specimen after the controlled thermal cycle, showing dark surface oxide-scale formation along the gauge section.

Representative optical images of the AISI 1045 specimens before and after the oxidation experiment are shown in Figure 2. These images illustrate the initial metallic surface and the visible surface oxidation developed after the controlled Joule-heating cycle.

The thermal images were captured using an Optris PI 450i digital infrared camera, equipped with an uncooled microbolometer focal plane array. The camera operates within a spectral range of 8–14 μm and features a noise-equivalent temperature difference (NETD) of 40 mK. The measurement range covers from −20 to 1500 °C. This thermal imaging system provides real-time raw radiometric data with a spatial resolution of 382 × 288 pixels at a frame rate of 80 Hz. Measurements were taken with a constant emissivity of 0.8 [15]. Accordingly, the thermographic values used in this study should be interpreted as apparent radiometric temperatures obtained under a constant-emissivity acquisition setting, rather than as fully emissivity-corrected true surface temperatures.

Although the infrared camera is capable of high-frame-rate acquisition, the dataset used for machine-learning analysis did not correspond to the complete raw video stream. Instead, thermographic records were exported using an approximately 5 s sampling interval, which was considered sufficient because the thermal evolution and oxidation-related surface changes occurred over time scales of seconds to minutes rather than milliseconds. In addition, thermograms acquired before the specimen reached the useful radiometric range or sufficient thermal contrast were excluded from the analysis. After temporal sampling and quality/range filtering, the final dataset contained 3356 valid thermographic records distributed as follows: 633 records from Experiment 1, 713 from Experiment 2, 641 from Experiment 3, 685 from Experiment 4, and 684 from Experiment 5.

### 2.2. Data Analysis

**Figure 3:**
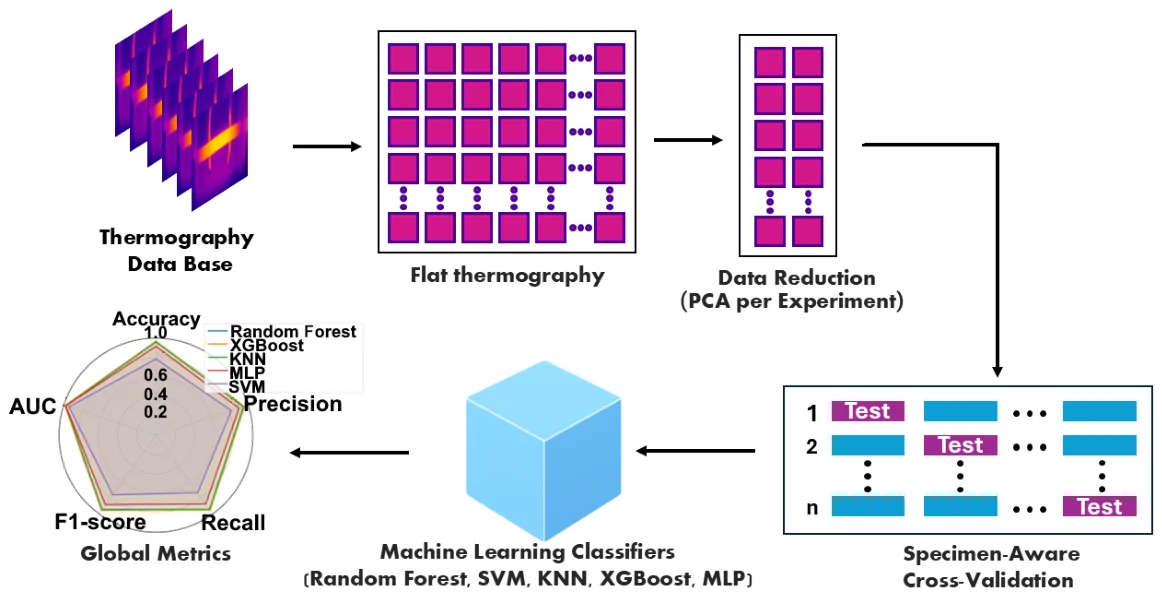
Workflow for Automatic Thermal Phase Classification from Infrared Thermography Data.

Figure 3 summarizes the workflow employed for the automatic classification of thermal phases from infrared thermography time series. Each thermographic frame acquired during the oxidation experiments was first transformed into a one-dimensional vector by flattening the original thermal matrix, preserving the complete radiometric information of the specimen surface.

To reduce dimensionality while preserving the dominant thermal evolution patterns, Principal Component Analysis (PCA) was applied independently to each experiment rather than globally across all experiments. This experiment-wise strategy was intentionally adopted to preserve the intrinsic thermal dynamics of each oxidation cycle and avoid imposing inter-experimental variance structures onto unseen specimens. Since the infrared camera remained spatially fixed throughout each experiment, the resulting thermographic sequences exhibited internally consistent spatial distributions and localized heterogeneity patterns, as previously observed in spatiotemporal thermographic analyses of AISI 1045 steel oxidation [7]. This configuration allowed PCA to capture the relative temporal evolution of the thermal field within each individual oxidation process rather than enforcing a global variance structure across independent experiments. It should be noted that the PCA scores obtained from each experiment were used as compact relative descriptors of the within-run thermographic evolution, rather than as absolute physical coordinates with identical eigenvectors across all experiments. Therefore, PC1 and PC2 should not be interpreted as strictly equivalent physical variables from one experiment to another. Their comparability was considered at the level of the reconstructed thermal-phase trajectories and was evaluated operationally through the specimen-aware Leave-One-Group-Out validation scheme. Under the controlled acquisition protocol, fixed camera position, consistent specimen geometry, and identical Joule-heating cycle, the first two principal components consistently summarized the dominant radiometric evolution of each run, allowing the supervised model to learn phase-related patterns across experiments.

**Figure 4:**
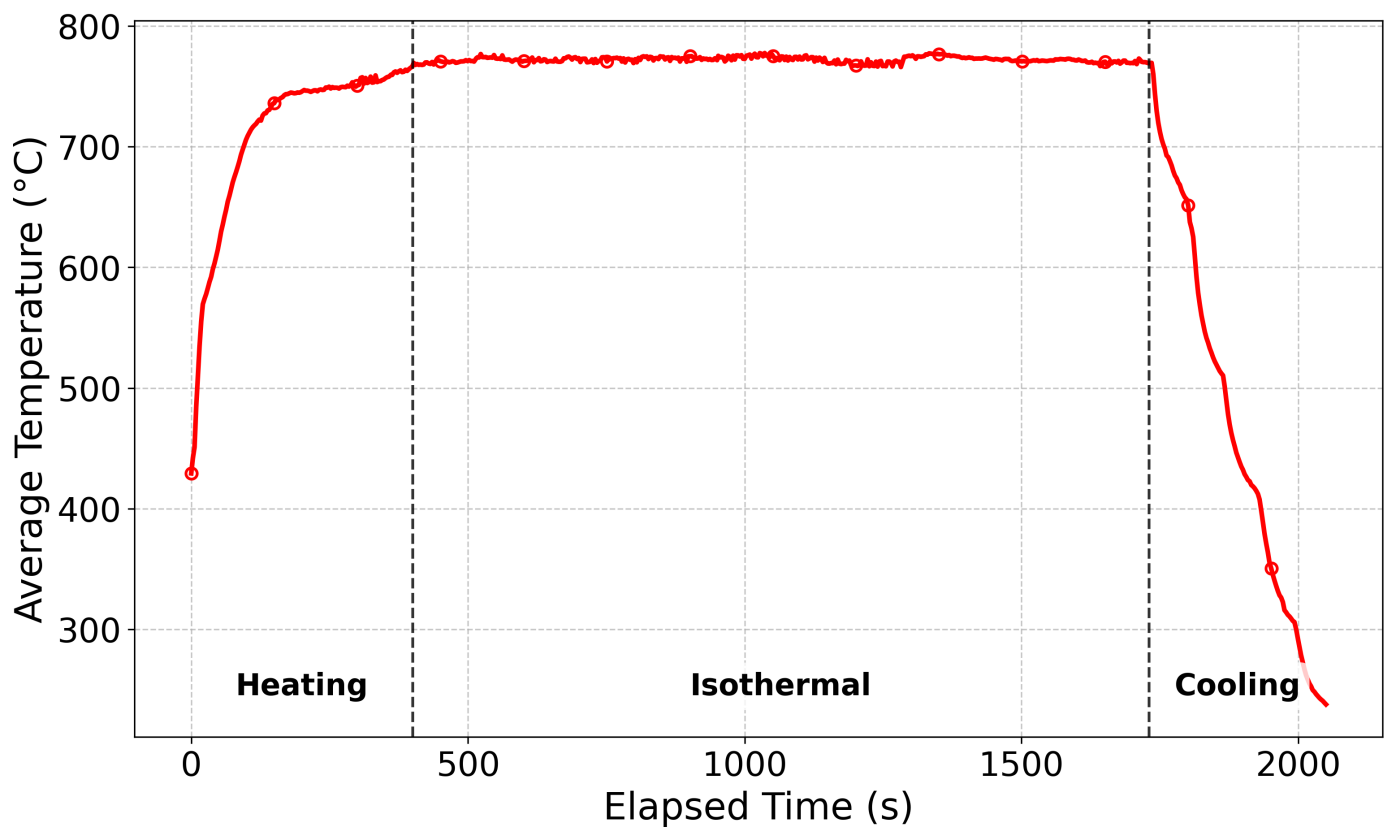
Representative average-temperature profile from Experiment 5 used to support the expert-guided assignment of heating, isothermal, and cooling labels during a Joule-heating oxidation experiment.

For each experiment, the flattened thermograms were projected onto the first two principal components (PC1 and PC2), generating a reduced latent representation used as input for the classification stage. The resulting PCA features from all experiments were subsequently organized into a unified dataset for supervised learning. PCA was implemented as an unsupervised, experiment-wise sequence representation rather than as a transformation fitted on pooled training experiments within each cross-validation fold. For each experimental run, only the flattened radiometric values of its thermograms were standardized and used to fit the corresponding PCA; thermal-phase labels were not included in either the standardization or PCA decomposition. Consequently, when an experiment was held out for testing, its PCA representation was derived solely from its own unlabeled thermographic sequence and was used only as test input. Neither the thermograms nor the PCA scores of the held-out experiment were used to fit the supervised classifier. Thus, experiment-level separation was preserved during supervised model training, while PCA served as an independent sequence-level descriptor of each oxidation run. A total of 3356 thermographic records were compiled from the five quality-controlled oxidation experiments. Each record was labeled according to three predefined thermal phases: heating (Class 0), isothermal exposure (Class 1), and cooling (Class 2). The final dataset comprised 798 samples for Class 0, 2028 samples for Class 1, and 530 samples for Class 2, reflecting the intrinsic temporal distribution of the oxidation cycle. The thermal-phase labels were assigned using an expert-guided criterion based on the experimental current program and the corresponding temperature–time evolution of each specimen. The applied current profile was used as the primary reference to identify the nominal heating, holding, and cooling intervals. However, because the specimen did not always reach a quasi-stationary thermal response immediately after the current reached its maximum value, the transition to the isothermal class was refined using the average temperature curves extracted from the thermographic sequence. Heating was defined as the interval characterized by the progressive current increase and a monotonic rise in the average specimen temperature. The isothermal exposure stage was assigned once the current remained at 160 A and the average temperature exhibited a quasi-stable plateau with only small temporal variations. Cooling was defined from the onset of the programmed current decrease and the corresponding sustained reduction in average temperature. The final transition points were reviewed and confirmed by metallurgical experts to ensure that the labels were physically consistent with the thermal response of each experiment. Figure 4 shows a representative average-temperature profile from Experiment 5, illustrating how the heating, isothermal, and cooling labels were assigned using the programmed current profile together with the observed thermal response.

The effectiveness of the thermal phase classification model was evaluated using five different supervised learning algorithms: Random Forest (RF), Multi-Layer Perceptron (MLP), Support Vector Machine (SVM), k-Nearest Neighbors (KNN), and Extreme Gradient Boosting (XGBoost). The comparative evaluation used predefined and reproducible model configurations rather than an exhaustive hyperparameter-optimization procedure, since the objective was to assess the feasibility of the PCA-based representation under a common validation protocol. The main settings were as follows: Random Forest with 100 trees, MLP with one hidden layer of 100 neurons and 500 maximum iterations, SVM with a radial basis function kernel and probability estimates enabled, KNN with five neighbors, and XGBoost using multiclass log-loss as the evaluation metric. A fixed random seed of 42 was used when applicable. The resulting PC1–PC2 features were standardized within the cross-validation pipeline, with the scaler fitted only on the training experiments of each fold and then applied to the corresponding held-out experiment. No oversampling, undersampling, or synthetic data generation was applied, because the class distribution reflected the physical duration of the heating, isothermal holding, and cooling stages. Instead, class imbalance was addressed during evaluation by reporting macro-averaged precision, recall, and F1-score, together with class-specific AUC values and confusion matrices. Each model was trained and tested using a rigorous Leave-One-Group-Out (Specimen-Aware) 5-fold cross-validation scheme to prevent temporal and inter-experimental data leakage and assess generalization to unseen specimens. The input features consisted of the first two principal components (PC1 and PC2) extracted from the flattened thermographic sequences of the five quality-controlled oxidation experiments. This methodology follows best practices for multivariate analysis and thermographic machine learning workflows [16,17].

## 3. Results

### 3.1. Latent Space Visualization and Data Separability

**Figure 5:**
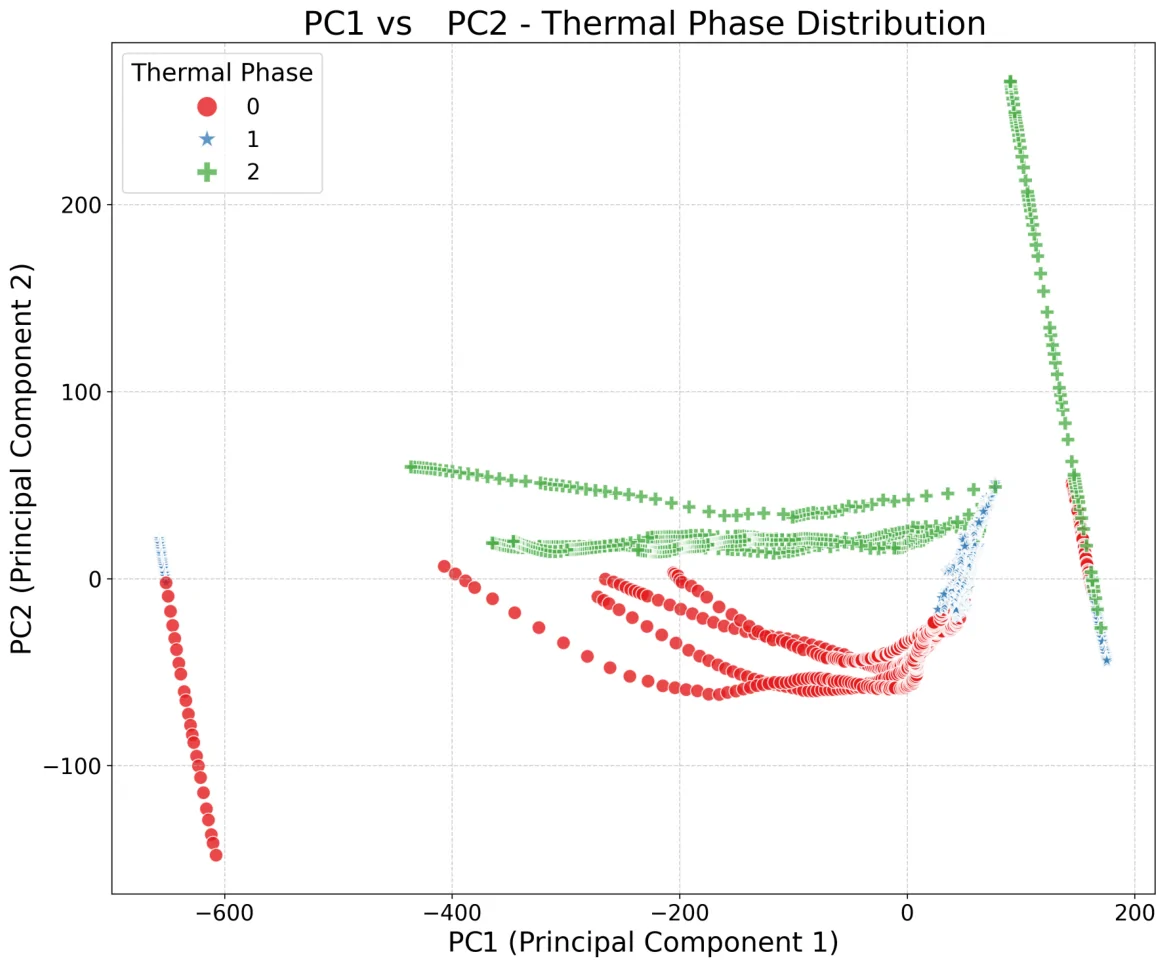
Projection of the experiment-wise PCA representations of the thermographic time series onto the two-dimensional latent feature space.

Prior to classifier training, we analyzed the geometric structure of the thermographic data within the reduced feature space. Figure 5 depicts the projection of the flattened thermograms onto the first two principal components (PC1 vs. PC2), colored by thermal phase.

The visualization reveals that the dataset does not form simple, linearly separable clusters, but rather follows continuous non-linear manifolds corresponding to the temporal evolution of the oxidation cycle. Notably, the heating (Class 0) and cooling (Class 2) phases follow distinct paths in the latent space, indicating that the PCA representation captures different thermal-radiometric trajectories across the experimental cycle. These trajectories may reflect the combined influence of temperature evolution, emissivity changes, and surface transformations occurring during oxidation; however, they cannot be attributed specifically to oxide-layer growth without independent structural or chemical characterization. This structural complexity suggests that non-linear classifiers (such as Neural Networks) may offer superior performance in decoding these phase transitions compared to linear decision boundaries.

The explained-variance analysis (shown in Table 1) supports the use of the first two principal components as a compact representation of the thermographic sequences. Across the five experiments, PC1 explained between 83.69% and 96.01% of the variance, whereas PC2 explained between 3.65% and 8.86%. Together, PC1 and PC2 retained more than 90% of the variance in all experimental runs, with a mean cumulative explained variance of 94.93 ± 2.96%. This result justified the selection of two principal components for the subsequent classification stage.

### 3.2. ML Model Performance

**Figure 6:**
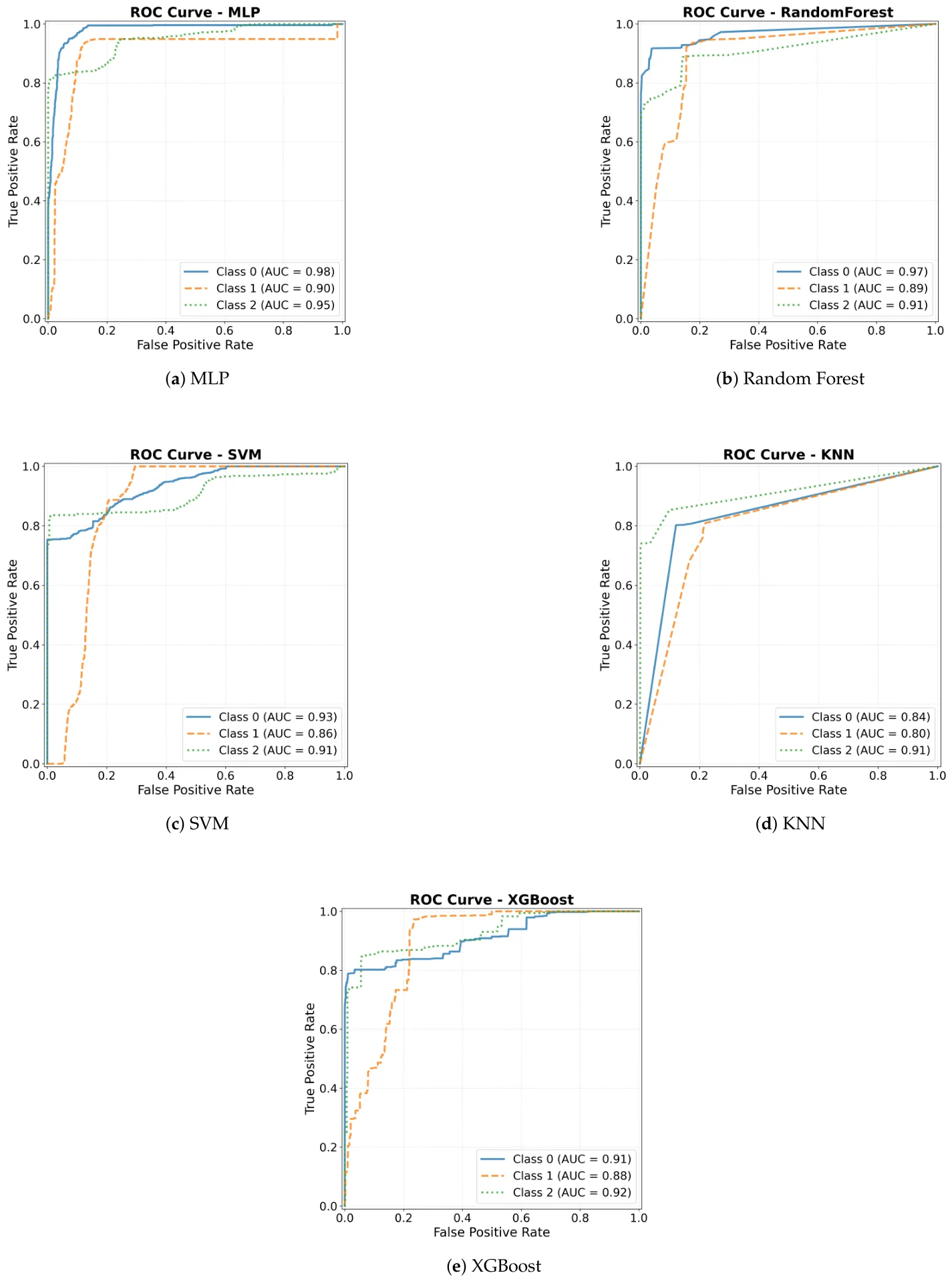
ROC curves for the five evaluated classifiers under Specimen-Aware cross-validation: (**a**) Multi-Layer Perceptron, (**b**) Random Forest, (**c**) Support Vector Machine, (**d**) k-Nearest Neighbors, and (**e**) XGBoost.

The classification performance obtained using the proposed experiment-wise PCA workflow and specimen-aware validation strategy is summarized in Table 2 and Figure 6. The evaluated machine learning models included Random Forest (RF), Multi-Layer Perceptron (MLP), Support Vector Machine (SVM), k-Nearest Neighbors (KNN), and Extreme Gradient Boosting (XGBoost).

Table 2 reports the mean and standard deviation of the performance metrics across the five Leave-One-Group-Out validation folds. Because each fold corresponded to one held-out experiment, the reported dispersion reflects inter-experimental variability rather than image-level uncertainty. Among the evaluated classifiers, the Multi-Layer Perceptron (MLP) achieved the highest average performance, with an accuracy of 0.8934 ± 0.0723 and a macro-F1 score of 0.8562 ± 0.1247. The remaining classifiers achieved average accuracies between 0.8023 and 0.8181, with larger fold-to-fold variability, indicating that model performance was affected by differences among individual experimental runs.

The individual fold-wise results are provided in Table A1. These results show that one held-out experiment produced lower classification performance for several models, highlighting the importance of reporting experiment-level variability and supporting the interpretation of the present study as a controlled proof of concept.

Compared to earlier techniques, this work achieves substantially higher accuracy in phase classification. Previous studies of steel microstructure or oxide detection using ML reported 60–83% accuracy under similar multi-class settings. For comparison, Hiremath et al. obtained 70% accuracy and a macro F1-score of 0.61 when classifying four microstructural phases in SEM images of commercial steels using texture features and machine learning [18]. In our experiments, the multilayer perceptron (MLP) model achieves 89.3% overall accuracy (Table 2), roughly a 10–20 percentage-point improvement over those benchmarks, indicating a meaningful advance. Furthermore, metrics such as the AUC values for heating, isothermal exposure, and cooling ( 0.98, 0.90, and 0.95, respectively) show that our classifier ranks thermal-phase instances very well even if some marginal misclassifications occur. The observed performance is consistent with the use of PCA-projected thermal-time features combined with a nonlinear model: the dimensionality-reduced trajectories preserve the dominant thermal-radiometric evolution observed during the oxidation experiments, while the MLP can model nonlinear patterns within this reduced feature space. In short, this approach appears to outperform simpler ensemble methods (e.g., Random Forest at 80.2% accuracy) and matches or exceeds accuracies reported in related steel monitoring tasks.

The classification results can be interpreted primarily in terms of the thermal-radiometric evolution of the programmed cycle. During heating, the specimen exhibits a progressive increase in apparent radiometric temperature, whereas the isothermal stage is characterized by a quasi-stable plateau and the cooling stage by a sustained decrease. Because the plateau and transition regions exhibit subtler thermal-radiometric changes, the MLP may benefit from its ability to model nonlinear decision boundaries in the PC1–PC2 feature space. These patterns occur during the oxidation process and may include contributions from emissivity evolution and surface changes, but the present data do not allow them to be assigned to specific oxide-growth regimes without complementary structural or chemical measurements.

The discriminative capability of the classifiers was further analyzed using Receiver Operating Characteristic (ROC) curves. Figure 6 compares the ROC profiles of all five evaluated classifiers under specimen-aware cross-validation.

The MLP model exhibited strong discriminative capability across the three thermal phases, with AUC values of 0.98 for heating (Class 0), 0.90 for isothermal exposure (Class 1), and 0.95 for cooling (Class 2). This high cooling AUC was computed from pooled out-of-fold predictions under specimen-aware cross-validation, in which each cooling sample was evaluated as part of a held-out experiment, and was therefore not derived from a small isolated test subset. These results indicate that the proposed model was particularly effective in distinguishing the heating and cooling stages, while the isothermal phase remained the most challenging due to its greater overlap with adjacent transition regions. The Random Forest classifier also showed good class-separation capability, reaching AUC values of 0.97, 0.89, and 0.91 for Classes 0, 1, and 2, respectively. However, the consistently higher AUC values of the MLP, together with its superior overall classification metrics, indicate that the neural network provided a more accurate discrimination of phase boundaries in the latent feature space. This behavior supports the suitability of the MLP as the best-performing model among the evaluated classifiers under the present experimental conditions.

**Figure 7:**
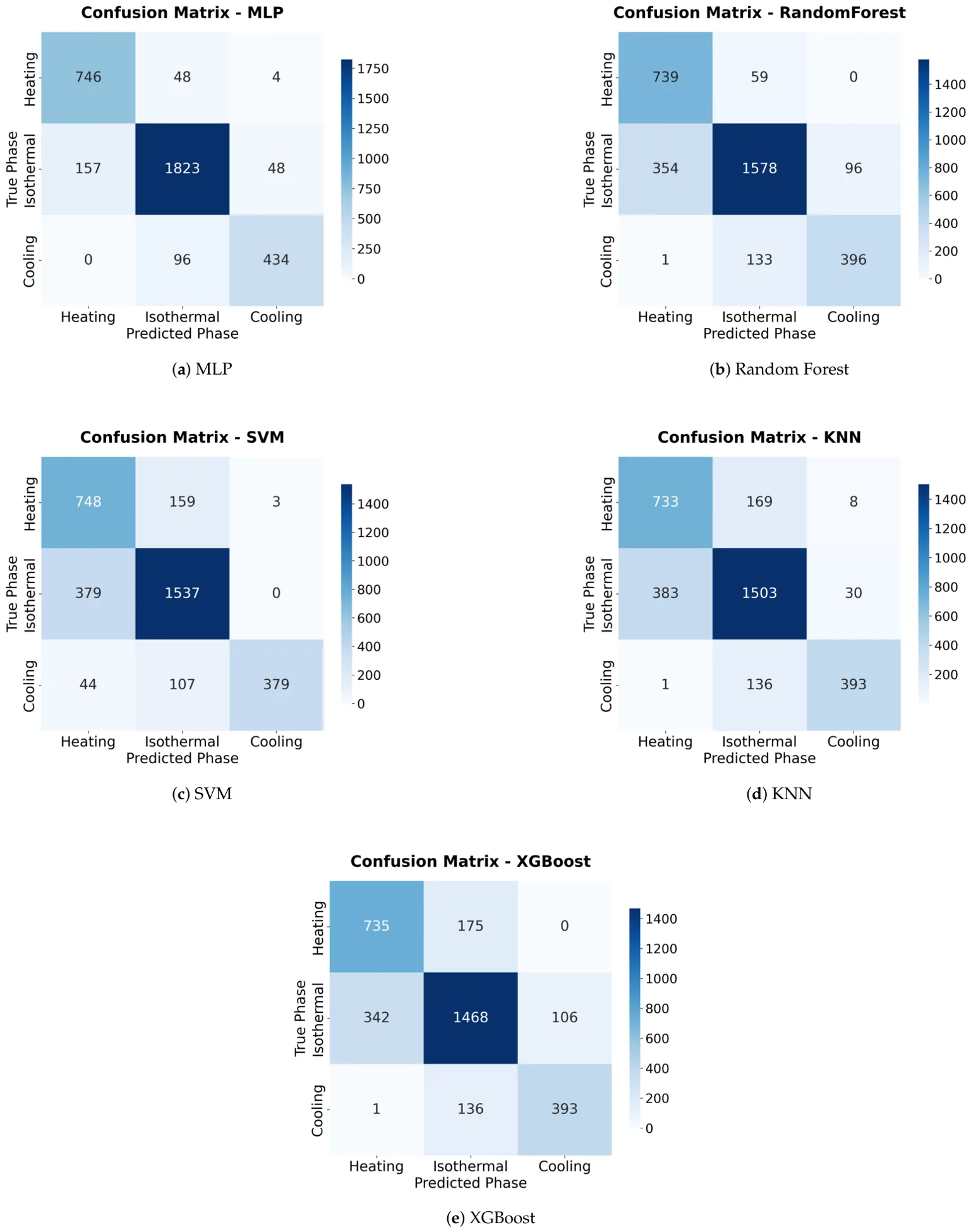
Confusion matrices obtained under Leave-One-Group-Out (Specimen-Aware) cross-validation for the five evaluated classifiers: (**a**) MLP, (**b**) Random Forest, (**c**) Support Vector Machine, (**d**) k-Nearest Neighbors, and (**e**) XGBoost.

Figure 7 compares the confusion matrices of the five evaluated classifiers under specimen-aware cross-validation. A critical observation is that both models achieve high thermodynamic consistency, with negligible misclassifications between the opposing phases of Heating and Cooling (Class 0 vs. Class 2). This confirms that the experiment-wise PCA representation preserved physically consistent thermal trajectories across oxidation phases despite being computed independently for each experiment.

However, the MLP showed superior performance in the classification of the Isothermal phase (Class 1). The Random Forest classifier (Figure 7b) exhibits a tendency to misclassify isothermal frames as heating (354 samples), suggesting a difficulty in detecting the stabilization of the thermal profile at the onset of the plateau. In contrast, the MLP (Figure 7a) significantly reduces this transition error, correctly identifying 1823 isothermal samples compared to 1578 for the Random Forest. This indicates that the neural network better captures the subtle non-linear radiometric variations associated with the quasi-stable isothermal stage, making it a more reliable classifier within the present thermal-phase recognition task.

Most classification errors occurred between temporally adjacent phases, particularly at the heating-to-isothermal and isothermal-to-cooling transitions, whereas direct confusion between heating and cooling was negligible. This error pattern is physically plausible because adjacent phases share gradual thermal and radiometric changes near their boundaries, making their precise temporal demarcation inherently ambiguous. The classifier therefore appears to preserve the overall thermodynamic progression of the oxidation cycle, with errors concentrated mainly at transition intervals rather than between physically opposed states.

## 4. Discussion

### 4.1. Interpretation and Implications

Steel oxidation at high temperature forms surface oxide scales that critically affect product quality and manufacturing efficiency. For instance, oxide scales formed during hot rolling can substantially affect the surface quality of the resulting products [19]. Removing or controlling these scales incurs cost and process complexity. Infrared thermography (IRT) offers a non-contact means to monitor surface temperature in real time over large areas, which is valuable for continuous process monitoring. In particular, IRT’s large-area, real-time imaging capabilities can detect subtle phase transitions during heating and cooling without interfering with the process [8]. Coupling IRT with automated analysis thus targets a major industrial need: ensuring oxidation is well-controlled to improve efficiency, safety and quality. Within this context, the correlation analysis provides additional insight into the physical interpretation of the principal-component representation used for thermal-phase classification. PC1 was mainly associated with the global thermal-radiometric evolution of the specimen, showing a strong positive correlation with the average temperature trajectory in all experiments (mean correlation: 0.9915 ± 0.0145). In contrast, PC2 showed a weak and variable correlation with average temperature (mean correlation: 0.0456 ± 0.1301), indicating that it captured residual information not directly explained by the global temperature trend. This residual component may reflect spatial heterogeneity, local emissivity changes, and oxide-layer-related radiometric variations. Therefore, the PCA representation should be interpreted as a compact thermal-radiometric descriptor of the oxidation sequence rather than as a direct measurement of oxide thickness or chemical phase composition. This interpretation is relevant for defining the scope of the proposed approach: the classifier operates on thermal-radiometric patterns associated with the oxidation cycle, rather than on direct chemical or microstructural measurements of the oxide scale. Moreover, because the average-temperature trajectory was used to refine the transition to the isothermal label and PC1 was strongly correlated with this same variable, a substantial portion of the observed classification capability is likely associated with the global thermal-radiometric progression of the experimental cycle. Therefore, the reported performance should not be interpreted as evidence that the classifier is primarily exploiting oxidation-specific surface signatures independently of the overall thermal evolution.

The constant-emissivity assumption is particularly relevant for interpreting what the classifier learned. Because oxide formation modifies surface emissivity during high-temperature exposure, the thermographic signal cannot be attributed exclusively to true temperature variations. Instead, the recorded radiometric patterns may reflect a combined contribution of specimen temperature, emissivity evolution, oxide-layer growth, and spatial surface heterogeneity. In this sense, the proposed model should be understood as classifying thermal-radiometric stages of the oxidation process rather than separating pure temperature effects from emissivity-driven effects. The strong correlation between PC1 and the average-temperature trajectory indicates that the dominant component of the classification is associated with global thermal evolution, whereas the weaker temperature correlation of PC2 suggests that residual radiometric information may include emissivity- and oxide-related surface changes.

This work has potential implications for automated process monitoring and Industry 4.0 applications. By enabling thermal-stage identification from thermographic imagery, the proposed approach could contribute to future decision-support tools based on live or near-real-time thermal data. For example, in a production-line scenario, a validated version of the workflow could assist in detecting premature or delayed onset of the thermal plateau associated with oxide-scale formation, providing information that may support alarms or process-adjustment strategies. Prior studies have noted that integrating IRT into process monitoring can enhance the reliability and operational efficiency of predictive maintenance systems, effectively serving as an online inspection sensor. In this context, the present results suggest that an MLP-based classifier may contribute to future Condition-Based Monitoring (CBM) strategies in steel processing by identifying deviations from expected thermal-stage progression. More broadly, this study demonstrates how combining PCA-based signal processing with machine learning can support thermal non-destructive testing and process-monitoring research. However, these industrial implications should be interpreted as prospective applications, since deployment in real production environments would require further validation under different steel grades, complex geometries, moving samples, variable emissivity conditions, and realistic industrial disturbances.

While accuracy and F1-score provide summaries of performance at the selected decision thresholds, the area under the ROC curve provides complementary information about class discrimination. The MLP model achieved exceptional AUC values, particularly for the heating phase (0.9791) and cooling phase (0.9474).

This discrepancy between high AUCs and moderate accuracy metrics (approx. 89%) is physically significant. It indicates that the classifier correctly assigns higher probabilities to the correct thermal phases (high discriminative power), but the misclassifications are concentrated solely at the temporal interfaces where the thermodynamic definition of a phase is inherently ambiguous. This error pattern may also be influenced by transition-boundary uncertainty during label assignment. Although the labels were not assigned from elapsed time alone, but from the programmed current profile, the average temperature evolution, and expert metallurgical review, the physical transition between heating, quasi-isothermal exposure, and cooling is gradual rather than instantaneous. Therefore, thermograms located near the heating-to-isothermal or isothermal-to-cooling boundaries may contain mixed radiometric characteristics from adjacent stages.

This overlap can be further explained by the coupled effects of thermal inertia, emissivity evolution, and oxide-scale development. At the heating-to-isothermal transition, the specimen temperature does not necessarily stabilize immediately after the applied current reaches its maximum value because the thermal response retains the influence of the preceding heating stage. Simultaneously, continued oxide-scale growth can modify the surface emissivity and therefore the recorded radiometric signal [5]. Conversely, during the isothermal-to-cooling transition, the thermal energy stored in the specimen delays the surface response to the programmed current reduction, while the developed oxide scale continues to influence surface radiation. Consequently, thermograms near both transition regions may exhibit radiometric characteristics shared by adjacent stages, increasing their overlap in the PCA feature space. In this context, some apparent misclassifications may reflect the inherent ambiguity of the transition intervals rather than strictly incorrect model predictions or inaccurate labeling. Future work could address this issue by incorporating transition classes, probabilistic labels, or derivative-based criteria from the temperature–time curves to better represent gradual phase boundaries.

From a deployment perspective, the high AUC suggests that the system’s sensitivity could be further optimized using methods such as Youden’s J statistic to adjust detection thresholds based on specific industrial requirements (e.g., prioritizing early detection of cooling over false positives), without retraining the underlying model structure. Thus, the probabilistic outputs appear to capture meaningful information about thermal-phase progression. However, their calibration and reliability should be formally evaluated before they are used for operational decision-making or real-time process control.

### 4.2. Limitations and Future Directions

The current model assumes consistent emissivity and laboratory-like conditions; in practice, variable emissivity or three-dimensional geometries could degrade accuracy. Therefore, the reported performance is specific to the controlled constant-emissivity acquisition protocol used in this study and should not be interpreted as emissivity-independent classification performance. Future work should incorporate emissivity calibration, multispectral thermography, or complementary structural measurements to separate temperature-driven and emissivity-driven contributions more explicitly. The method has been tested on a specific steel grade and heating profile, so its generality to other steels or industrial processes must be validated. In particular, changes in steel composition, oxide-scale chemistry, specimen geometry, surface curvature, thermal mass, and heat-transfer conditions may modify the thermographic trajectories learned by the model and reduce its transferability to other materials or geometries. Also, while PCA + ML yields good performance, it does not provide direct chemical identification of the oxide phases. Accordingly, the present workflow cannot discriminate among individual oxide species or distinct oxide-scale constituents. Any oxide-related contribution is embedded indirectly in the thermal-radiometric signal through changes in emissivity, surface condition, and thermal response, but it cannot be assigned to a specific chemical phase without complementary phase-resolved characterization.

In addition, because PCA was computed independently for each experiment, possible differences in component orientation, sign, and scaling may affect direct comparability across new experimental runs. Although the present specimen-aware validation indicates that the resulting trajectories were sufficiently consistent for the controlled dataset, future implementations should evaluate reference-PCA calibration, component alignment, or incremental PCA to improve robustness in batch and online settings. For a new experimental run under the same acquisition protocol, the same preprocessing workflow would be applied to the thermographic sequence, and the resulting PC1–PC2 score trajectory would be submitted to the trained classifier for thermal-phase assignment. This corresponds to a sequence-based implementation, where the thermographic evolution of the new run is available. Fully online frame-by-frame deployment would require a reference PCA basis calibrated from representative experiments, an incremental PCA strategy, or a component-alignment procedure to ensure consistent projection of new thermograms. In continuous annealing or other continuously operated industrial lines, where a complete experimental sequence may not be available before classification, experiment-wise PCA in its present form would therefore not be directly applicable; instead, a fixed reference PCA basis, incremental PCA, or an adaptive window-based projection strategy would be required for online thermal-stage monitoring.

Although PC1 and PC2 retained more than 90% of the variance in all experiments, higher-order components may still contain subtle radiometric information associated with localized surface changes or gradual phase-transition boundaries. Therefore, discarding these components may have contributed to residual misclassifications near adjacent thermal stages. Future work should evaluate the effect of including additional principal components or using variance-threshold-based component selection to determine whether higher-order features improve transition detection without increasing overfitting. Because all classifiers were evaluated using the same two-dimensional PC1–PC2 feature space, the comparison reflects their performance within a common compact representation rather than their maximum achievable performance with model-specific feature sets. Consequently, the intrinsic feature-selection capabilities of tree-based models such as Random Forest and XGBoost were not fully exploited in the present study. Future work should evaluate additional principal components, thermal descriptors, or image-based features to assess whether these models benefit from richer input spaces.

First, future studies should combine thermographic monitoring with phase-specific techniques, such as Raman spectroscopy or X-ray diffraction, because multimodal infrared and structural measurements have demonstrated that these techniques provide complementary information on phase evolution in metal oxides [20]. Second, expand the dataset to different materials and geometries, including 3D shapes and moving processes, to ensure robustness in field conditions. The choice of PCA combined with conventional machine-learning classifiers was motivated by the proof-of-concept nature of the study and by the limited number of independent experimental runs. Although convolutional or recurrent neural networks could directly learn spatial and temporal features from thermographic sequences, such end-to-end models typically require larger numbers of independent experiments, more extensive hyperparameter tuning, and higher computational resources to reduce the risk of overfitting. In contrast, the PCA-based representation provided a compact, reproducible, and interpretable feature space that retained more than 90% of the variance in all experimental runs while allowing a controlled comparison of classifiers under specimen-aware validation.

Accordingly, the present study does not establish PCA + MLP as a globally optimal architecture, but rather identifies MLP as the best-performing classifier among the classifiers evaluated within the common two-component PCA representation. Future studies should explore end-to-end deep learning architectures, such as temporal CNNs, recurrent networks, or hybrid CNN–RNN models, once larger and more diverse thermographic datasets become available. More broadly, a comparative benchmark should evaluate the present PCA-based workflow against fold-wise global PCA, richer principal-component representations, and handcrafted or learned thermal descriptors using a larger number of independent experimental runs. Finally, we recommend implementing this system in a feedback loop for process control: for example, using Youden’s J statistic or similar to choose optimal decision thresholds in real time, thereby enhancing sensitivity to critical transitions. By addressing these points, future studies can make the approach a practical tool for predictive maintenance and improved safety in thermal processing.

## 5. Conclusions

This study evaluated an experiment-wise PCA and machine learning workflow for the automatic recognition of heating, isothermal exposure, and cooling stages during controlled high-temperature oxidation experiments on AISI 1045 steel. Infrared thermography time series from five independent Joule-heating experiments were transformed into compact PCA-based representations and evaluated under specimen-aware leave-one-group-out cross-validation, thereby preserving experiment-level independence during model assessment. Among the evaluated classifiers, the Multi-Layer Perceptron achieved the strongest overall performance, with an accuracy of 0.8934 and an F1-score of 0.8562. The model also showed high class-discrimination capability, reaching an AUC of 0.95 for the cooling phase. Notably, classification errors were predominantly localized at the temporal boundaries between adjacent phases, reflecting the inherent thermodynamic ambiguity of these transitions rather than fundamental misclassifications between opposed physical states. This physically consistent behavior suggests that the neural network captured subtle non-linear radiometric variations associated with the thermal evolution of the oxidation process. From a practical standpoint, this data-driven approach provides a controlled proof of concept for thermography-based thermal-stage recognition. The ability to segment continuous thermographic acquisitions into discrete process states suggests a potential non-invasive pathway for future monitoring applications. However, the present results should not be interpreted as a fully validated industrial process-control solution, since validation was limited to one steel grade, simple cylindrical specimens, controlled Joule heating, and laboratory acquisition conditions. In addition, because PCA was computed independently for each experimental run, the present implementation is sequence-based and is not directly applicable to continuous online frame-by-frame monitoring. Such deployment would require a common projection strategy, such as a reference PCA basis calibrated from representative process data, incremental PCA, or an equivalent adaptive projection approach. The current methodology is also subject to certain limitations that define the scope for future work. The experimental validation was conducted under highly controlled laboratory conditions with assumptions of constant emissivity, evaluating a specific steel grade and simple cylindrical geometries. Furthermore, while the PCA-based approach successfully tracks thermal-radiometric evolution, it does not provide direct chemical identification of the evolving oxide scales. Future research should address these constraints by validating the models under variable emissivity conditions, different steel grades, more complex geometries, and moving-process scenarios; exploring end-to-end deep learning architectures capable of learning spatial-temporal features directly from thermographic sequences; and correlating thermal signatures with phase-specific structural techniques such as Raman spectroscopy or X-ray diffraction. Nevertheless, the results support the potential of thermography-based machine learning as a non-contact component of future monitoring systems for high-temperature steel processing.

## Figures and Tables

**Table 1:** Explained variance of the first two principal components (PC) and their correlation with the average specimen temperature for each experiment.

Experiment	PC1 (%)	PC2 (%)	PC1 + PC2 (%)	Corr. PC1–Temp.	$R^{2}$ PC1–Temp.	Corr. PC2–Temp.	$R^{2}$ PC2–Temp.
Experiment 1	96.01	3.65	99.66	0.9657	0.9326	0.2594	0.0673
Experiment 2	89.51	6.50	96.01	0.9966	0.9932	−0.0805	0.0065
Experiment 3	83.69	8.86	92.55	0.9982	0.9964	0.0520	0.0027
Experiment 4	88.30	4.88	93.18	0.9986	0.9972	0.0273	0.0007
Experiment 5	88.26	5.00	93.25	0.9986	0.9972	−0.0303	0.0009
Mean ± SD	89.15 ± 4.43	5.78 ± 2.00	94.93 ± 2.96	0.9915 ± 0.0145	0.9833 ± 0.0284	0.0456 ± 0.1301	0.0156 ± 0.0290

**Table 2:** Global performance metrics for all models using Specimen-Aware (Leave-One-Group-Out) 5-fold cross-validation. Results are reported as mean ± standard deviation across the five validation folds. Best overall performance shown in **bold**.

Model	Accuracy	Macro-Precision	Macro-Recall	Macro-F1
**MLP**	**0.8934 ± 0.0723**	**0.8740 ± 0.0994**	**0.8938 ± 0.0833**	**0.8562 ± 0.1247**
SVM	0.8181 ± 0.2473	0.8898 ± 0.1291	0.8289 ± 0.2096	0.7929 ± 0.2912
KNN	0.8097 ± 0.2291	0.8005 ± 0.2403	0.8246 ± 0.2025	0.7765 ± 0.2822
XGBoost	0.8026 ± 0.2431	0.7891 ± 0.2661	0.8223 ± 0.2108	0.7742 ± 0.2906
Random Forest	0.8023 ± 0.2426	0.7860 ± 0.2608	0.8252 ± 0.2101	0.7733 ± 0.2882

## Data Availability

The original data presented in the study are openly available in Zenodo at https://doi.org/10.5281/zenodo.20294022.

## Appendix A

**Table A1:** Individual fold-wise performance metrics for each classifier under Specimen-Aware Leave-One-Group-Out 5-fold cross-validation. Each fold corresponds to one held-out experimental run.

Model	Fold	Accuracy	Macro-Precision	Macro-Recall	Macro-F1
Random Forest	1	0.3207	0.2659	0.4112	0.2000
Random Forest	2	0.9547	0.9344	0.9712	0.9508
Random Forest	3	0.9563	0.9366	0.9638	0.9480
Random Forest	4	0.8934	0.8817	0.9054	0.8859
Random Forest	5	0.8864	0.9115	0.8747	0.8819
MLP	1	0.7678	0.6981	0.7494	0.6219
MLP	2	0.9766	0.9698	0.9828	0.9756
MLP	3	0.9158	0.8483	0.9466	0.8759
MLP	4	0.9401	0.9661	0.9348	0.9467
MLP	5	0.8668	0.8875	0.8553	0.8608
SVM	1	0.3302	0.6383	0.4189	0.2164
SVM	2	0.9722	0.9674	0.9734	0.9695
SVM	3	0.9844	0.9819	0.9731	0.9773
SVM	4	0.9358	0.9639	0.9249	0.9403
SVM	5	0.8682	0.8973	0.8540	0.8611
KNN	1	0.3539	0.3206	0.4243	0.2139
KNN	2	0.9518	0.9409	0.9696	0.9521
KNN	3	0.9454	0.9225	0.9358	0.9270
KNN	4	0.9109	0.9072	0.9188	0.9075
KNN	5	0.8864	0.9115	0.8747	0.8819
XGBoost	1	0.3207	0.2596	0.4072	0.1969
XGBoost	2	0.9620	0.9450	0.9736	0.9582
XGBoost	3	0.9563	0.9537	0.9568	0.9540
XGBoost	4	0.8920	0.8803	0.9021	0.8838
XGBoost	5	0.8822	0.9069	0.8717	0.8780
